# Expression of platelet-derived growth factor in the vascular walls of patients with lower extremity arterial occlusive disease

**DOI:** 10.3892/etm.2015.2275

**Published:** 2015-02-09

**Authors:** YANG ZHANG, WANG-DE ZHANG, KE-QIN WANG, TAN LI, SHENG-HAN SONG, BIAO YUAN

**Affiliations:** Department of Vascular Surgery, Beijing Chaoyang Hospital, Capital University of Medical Sciences, Chaoyang, Beijing 100020, P.R. China

**Keywords:** arteriosclerosis obliterans, platelet-derived growth factor, quantitative polymerase chain reaction

## Abstract

The aim of the present study was to quantitatively analyze the expression of platelet-derived growth factor (PDGF)-A and PDGF-B in the vascular walls of patients with lower extremity arterial occlusive disease (LEAOD). The expression levels of PDGF-A and PDGF-B in the lower extremity arteries of 19 LEAOD patients (case group) and three healthy subjects (control group) was determined using quantitative polymerase chain reaction. Intergroup comparisons revealed that the relative mRNA expression levels were higher in the case group, as compared with the control group, for PDGF-A (34.38±5.80 vs. 21.94±1.05; P<0.05) and PDGF-B (33.95±5.92 vs. 24.15±3.12; P<0.05). In addition, the expression of PDGF-A revealed a positive linear correlation with the expression of PDGF-B (P<0.05). Therefore, the expression levels of PDGF-A and PDGF-B were found to be higher in the vascular walls of LEAOD patients, while the expression of PDGF-A was found to correlate with the expression of PDGF-B. A significant increase in the expression levlels of PDGF-A and PDGF-B were observed in the vascular walls of patients with LEAOD, and the expression of PDGF-A was associated with the expression of PDGF-B.

## Introduction

Atherosclerosis (AS) and its complications are the leading cause of mortality worldwide. The proliferation of medial smooth muscle cells (SMCs) plays a key role in the pathogenesis of AS. Macrophages from intimal SMCs accumulate in the subendothelial region, resulting in the formation and rupture of plaques, and consequent vascular occlusion and clinical events (1,2).

Previously, a serum-derived factor in platelets was found to be able to stimulate the growth of SMCs; platelet-derived growth factor (PDGF) was subsequently purified. PDGF can accumulate at injury sites and proliferate in the extracellular matrix, expanding the area of injury and/or leading to further damage. PDGF plays important roles in different stages during the development and progression of various diseases. The findings of a number of studies have supported the hypothesis that PDGF is associated with SMC accumulation during the development of AS (1,3).

Lower extremity arterial occlusive disease (LEAOD) is a manifestation of systemic AS in the limbs. The growth of atherosclerotic material and secondary thrombosis can lead to stenosis or occlusion of the arterial lumen, which is clinically manifested as a limb blood circulation disorder that may predispose patients to ulcers or gangrene. While the majority of previous studies have been performed on animals or cells *in vitro* (4–8), the current study was conducted using the diseased arteries of LEAOD patients. The expression levels of PDGF-A and PDGF-B were analyzed in the AS plaques of LEAOD patients in order to investigate their association with the development of AS.

## Materials and methods

### Laboratory materials

Arterial specimens were collected from 19 LEAOD patients (case group; male, 14; female, 5; age, 51–70 years) who had undergone an above-knee amputation between September 2007 and September 2010. All the LEAOD cases were classified as Fontaine stage IV (9), suffering from various stages of ischemia and necrosis in the limbs. Comorbidities included hypertension (n=14), hyperlipidemia (n=4), coronary heart disease (n=11), diabetes (n=11) and uremia (n=1). In addition, normal artery samples from three patients (male, 2; female, 1; age, 25–52 years) who had undergone an above-knee amputation for reasons not associated with AS, had no atherosclerosis, tumors or Takayasu’s ateritis, were used as the control group. Specimen collection was examined and approved by the Ethics Committee of the Capital University of Medical Sciences (Beijing, China), and signed informed consent was obtained from each patient. The specimens were stored in liquid nitrogen at −80°C during transportation.

### Reagents and equipment

Reagents used in the study are listed in Table I, while the equipment used are listed in Table II. Other chemical agents, including diethylpyrocarbonate (DEPC)-treated water, chloroform, anhydrous ethanol, isopropanol and EDTA, were purchased from Sinopharm Chemical Reagent Co., Ltd., (Shanghai, China).

### Specimen treatment

Following adventitia of the cryopreserved artery, the internal median layers, weighing 0.20 g, were harvested from the case and control groups.

### Total RNA extraction (TRIzol method)

TRIzol reagent (1 ml) was added to the harvested arterial specimens. The specimens were ground, homogenized for 15 sec and incubated at room temperature for 5 min. Next, 0.2 ml chloroform was added and the tubes were shaken vigorously for 15–30 sec by hand, followed by incubation at room temperature for 5 min. Subsequently, the samples were centrifuged at 12,000 × g for 15 min at 4°C. The upper aqueous phase, containing the total RNA, was carefully removed and transferred to a new 1.5-ml centrifuge tube. Isopropyl alcohol (0.5 ml) was added and the samples were incubated at room temperature for 10 min following thorough mixing. Next, the samples were centrifuged at 12,000 × g for 8 min at 4°C. The supernatant was carefully removed, and 1 ml DEPC-treated water (75%) was added slowly along the tube sides. The samples were mixed thoroughly and incubated for 30 min. Centrifugation (8,000 g) at 4°C for 5 min was performed to discard the supernatant, after which the samples were air-dried at room temperature for 5–10 min. The pellet was dissolved in 20 μl DEPC-treated water. For electrophoresis, the samples (5 μl) were added to a 1.5% agarose gel (10 V/cm) and were run for ~20 min. The gels were analyzed using a UV spectrophotometer. Subsequently, RNA was reverse-transcribed to provide cDNA.

### cDNA synthesis

The reaction solution shown in Table III was prepared in a 0.2-ml centrifuge tube. The reaction was stopped by heating at 65°C for 5 min, followed by cooling on ice. The materials listed in Table IV were added in the indicated order. Reverse transcription was subsequently performed, according to the conditions listed in Table V. cDNA samples were stored at −20°C until required for further use.

### Quantitative polymerase chain reaction (PCR)

Primers for PDGF-A and PDGF-B used in the reaction system are listed in Tables VI and VII, respectively. The target genes and the internal control (β-actin) were amplified separately. The reaction system is shown in Table VIII. The reaction conditions were as follows: 95°C for 3 min, 40 cycles: 95°C for 15 sec, 60°C for 15 sec; 72°C extension for 3 min. Following the reaction, melting-curve analysis was performed to verify the product specificity.

When a fluorescent dye is bound to the double-stranded DNA, a particular sequence cannot be identified, potentially leading to false-positive results. Therefore, for product identification, melting-curve analysis was performed to eliminate the impact of the primer dimers. For each sample, a single peak on the melting-curve indicated a single PCR product.

For quantitative analysis, the target gene mRNA expression was calculated using the following formula: 2^−(Ct of cytokine − Ct of β−actin)^×10^3^, where Ct represented the cycle threshold (fractional cycle number at which the amount of amplified copies reaches a fixed threshold). Fewer cycles are required to reach exponential amplification when the starting copy number of the target is high; thus, the Ct value is smaller. The cytokines used in the current study were PDGF-A and PDGF-B.

### Statistical analysis

Ct values of PDGF-A and PDGF-B were used as the statistical parameters. Statistical analysis was performed using SPSS version 16.0 software (SPSS, Inc., Chicago, IL, USA). Data in the case and control groups were analyzed using the t-test or the t′-test based on homegeniety of variance. In the case group, pairwise correlations were investigated using linear correlation analysis.

## Results

### Ct values of the cytokines

The Ct values of PDGF-A and PDGF-B in the case and control groups are summarized in Tables IX and X, respectively. The homogeneity tests revealed that P>0.05 in each group.

### Comparison of the Ct values

**Figure 1:**
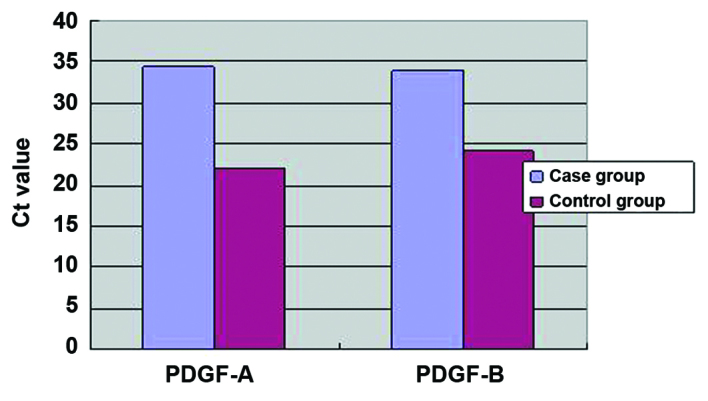
Ct values of PDGF-A and PDGF-B in the case and control groups. PDGF, platelet-derived growth factor.

Comparisons of the Ct values for PDGF-A and PDGF-B between the case and control groups are shown in Fig. 1. A statistically significant difference was observed for the PDGF-A Ct values between the case and control groups (t′=8.51, P=0.000). In addition, the Ct value of PDGF-B was found to be significantly different between the two groups (t′=2.77, P=0.012). Therefore, the expression levels of PDGF-A and PDGF-B were found to be significantly higher in the case group when compared with the control group.

### Correlation analysis

**Figure 2:**
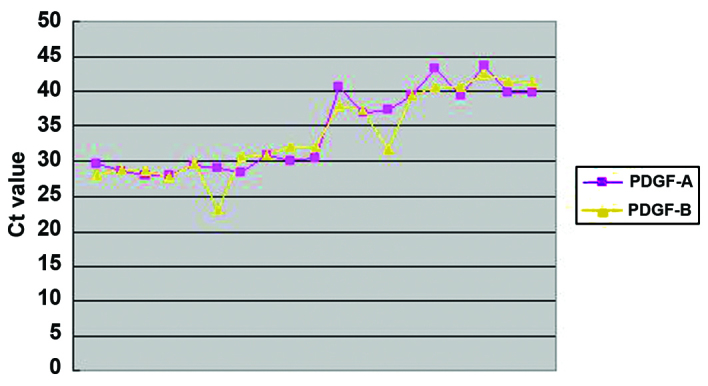
Correlation between the expression levels of PDGF-A and PDGF-B in the vascular walls of the case group. PDGF, platelet-derived growth factor.

The correlation between the expression levels of PDGF-A and PDGF-B in the case group is shown in Fig. 2. The Ct value of PDGF-A was significantly correlated with the Ct value of PDGF-B (r=0.918, P=0.000), indicating that the expression of PDGF-A in the vascular wall correlates with the expression of PDGF-B.

## Discussion

The PDGF family includes a number of PDGFs and PDGF receptors (PDGFRs). Mitogenic PDGF is widely distributed throughout the body and produced in platelets; however, the growth factor can also be produced in SMCs, macrophages and endothelial cells (ECs) following vascular injury (10). The PDGF family consists of disulfide-bonded homodimers or heterodimers with four possible subunits (PDGF-A, PDGF-B, PDGF-C and PDGF-D). The A-chain is typically synthesized and secreted by SMCs that are proliferated following intimal injury, whereas the B-chain is produced mainly by macrophages and ECs (1). After production, PDGF activates the PDGFR via autocrine and/or paracrine signaling. Two distinct subunits, α and β, dimerize to form three types of PDGFR (PDGFR-αα, -αβ and -ββ).

As a key mediator of inflammation, PDGF can induce the migration and proliferation of SMCs in the intima, which is an important mechanism in the development of AS. PDGF exhibits a number of biological characteristics. Firstly, PDGF has a mitogenic effect, whereby PDGF facilitates DNA synthesis and enables cell lysis and proliferation by adjusting the regeneration of extracellular matrix, which stimulates the division and proliferation of vascular SMCs (VSMCs) (11). Wang *et al* (12) demonstrated that PDGF-B has stronger proproliferative and mitogenic effects compared with PDGF-A. Secondly, PDGF exhibits chemotactic activity by contributing to SMC and fibroblast accumulation at injured sites, which is subsequently followed by the proliferative response. This chemotactic activity is particularly important for injury repair. Finally, PDGF exhibits a vasoconstrictive effect (13).

SMCs are the direct cause of vascular stenosis or occlusion. As a key inflammatory mediator, PDGF-A is synthesized and secreted by SMCs in the vascular intima during injury, while PDGF-B is produced mainly by macrophages and ECs (14). PDGF-A and PDGF-B increase the thickness of the fibrous capsules of plaques (15). Various inflammatory signaling pathways participate in the development of AS. As the most potent growth factor of SMCs, PDGF-B induces the synthesis and secretion of PDGFR-β and SMC progenitor cells, causing intimal thickening (16,17). Furthermore, PDGF-B induces the recruitment and differentiation of intimal SMCs and stimulates angiogenesis (18).

PDGF-AA, PDGF-AB and PDGF-BB are potent promoters of VSMC proliferation and their migration in the subendocardial region. The expression level of PDGF can increase by 10–20 times at 6 h following vascular injury. During the development of AS, PDGF stimulates the medial SMCs to change from a contractile to a synthetic phenotype, increasing their proliferative capacity. Following the phenotypic change, the cells obtain stronger synthesis and secretory functions. After proliferation, the intimal SMCs express PDGF-A and PDGFR genes and secrete bioactive PDGF-like molecules. The bioactive PDGF-like molecules can promote the proliferation and migration of VSMCs and the deposition of the extracellular matrix (19). In addition, PDGF-A and PDGF-B are involved in the thickening of the fibrous capsules of plaques (15), and may detach from the fat-rich core matrix of thrombi in the arterial lumen, further exacerbating the clinical symptoms (14).

A large number of studies (20,21) have demonstrated increased expression levels of PDGF and PDGFR in human AS plaques, coronary blood vessels following angioplasty and the vascular walls of animal models with vascular injury. These observations indicate that PDGF and PDGFR are involved in the pathogenesis of AS and the repair of arterial injury.

To date, the majority of studies have been conducted on human medium-sized arteries or on arteries of animal models; however, the present study was performed using human lower limb large arteries. Quantitative analysis of the expression levels of PDGF-A and PDGF-B in the diseased arteries revealed that the two cytokines were highly expressed in the case group. Therefore, high expression levels of PDGF-A and PDGF-B are also present in the AS plaques of the human lower limb large arteries. Furthermore, a previous study demonstrated that PDGF can directly stimulate the expression and enhance the activity of nuclear factor-κB (NF-κB), which is a key regulator of arteriosclerosis (26). The highly-expressed PDGF may enter the vascular subendothelium and activate NF-κB in the vascular SMCs, enhancing cellular proliferation.

Each PDGF subtype exhibits angiogenic activity. Arterial occlusion resulting from excessive neointimal proliferation is a sign of restenosis following balloon angioplasty in AS patients. Inhibition of intraplaque angiogenesis is an important approach used in the stabilization of AS plaques. A number of growth factors and cytokines are involved in the process of restenosis, among which PDGF is considered to be the most significant, due to the effect of PDGFRβ on the tunica media SMCs. In addition, various PDGF-induced cell responses, including the regulatory effect of PDGF on vascular tone, the negative feedback-driven regulation during platelet activation and the promotion of neovascularization, are associated with AS diseases. Previous studies have also investigated the expression of PDGF-A and PDGF-B in arteries following coronary angioplasty. Over past two decades, a number of studies have investigated the stent-based delivery of the PDGFR inhibitor; however, its effectiveness remains unsatisfactory. Furthermore, inhibition of PDGF expression may inhibit injury-induced intimal expansion and the formation of collateral circulation, hampering the improvement of the ischemic status. Therefore, future studies should utilize the existing knowledge regarding PDGF and its receptor in order to facilitate the prevention and treatment of AS and the resulting clinical events (22,23).

The results of the present study confirmed the positive linear correlation between highly-expressed PDGF-A and PDGF-B in the lower limb arteries of LEAOD patients. In addition, a PDGFR kinase inhibitor-coated stent was found to partially inhibit vascular restenosis following percutaneous transluminal coronary angioplasty, also indicating that PDGF-A and PDGF-B are closely correlated with the development of AS (24,25). Kozaki *et al* (27) demonstrated that the elimination of PDGF-B in circulating cells and the blockade of two PDGFRs transiently delayed fibrous cap formation in rats with early AS. However, SMC accumulation at the injured sites was not prevented, indicating that stenosis or occlusion of the arterial lumen by AS is a complex process involving numerous factors.

PDGF-A and PDGF-B play important roles in various stages of AS. Therefore, future research should focus on blocking their adverse effects on arteriosclerosis, while preserving their positive roles in countering arteriosclerosis and improving ischemia.

The present study had several limitations. Firstly, the specimens used in the study were obtained from the arterial vessels of patients with advanced arteriosclerosis; thus, the samples were unable to fully reflect the associations between PDGF-A, PDGF-B and arteriosclerosis throughout the disease course. Furthermore, although the specimens were obtained from LEAOD patients, the disease course differed markedly among the patients (between four days and 10 months). Complications included hypertension, hyperlipidemia and diabetes, which are independent risk factors of arteriosclerosis. As a result, the expression levels of PDGF-A and PDGF-B may have differed due to differences in the severity of the disease. In addition, the storage period of the arterial vessels differed among the samples, with intervals up to three years. Since RNA degrades easily, the long storage interval may have affected the results of the current study. In future studies, specimens should be harvested, transported and stored in a more consistent manner. Furthermore, based on the pathological stages and risk factors of arteriosclerosis, the expression levels of PDGF-A and PDGF-B in vascular walls should be analyzed during different stages of arteriosclerosis in order to provide solid clinical evidence for the prevention and treatment of the arteriosclerosis obliterans. In concludion, the results of the present study demonstrated that the levels of PDGF-A and PDGF-B were increased in the vessel wall of patients with LEAOD.

## Figures and Tables

**Table I:** Reagents used in the study.

Reagent	Supplier
Total RNA extraction reagent, TRIzol	Invitrogen Life Technologies, Carlsbad, CA, USA
Agarose (1.5%)	BioWest, Barcelona, Spain
2X Power *Taq* PCR MasterMix	BioTeke Corporation, Beijing, China
Primers Sangon	Biotech Co., Ltd., Shanghai, China
ReverTra Ace-α-^®^ cDNA kit	Toyobo Co., Ltd., Osaka, Japan
DEPC water	Beyotime Institute of Biotechnology, Haimen, China
Multi-functional DNA Gel Extraction kit II (Spin-Column)	BioTeke Corporation, Beijing, China
Realtime PCR Master Mix	Toyobo Co., Ltd., Osaka, Japan

**Table II:** Equipment used in the study.

Equipment	Supplier
Ultraviolet spectrophotometer	BioVision, Milpitas, CA, USA
Low-temperature refrigerator	Sanyo Electric Co., Ltd., Moriguchi, Japan
Low-temperature high-speed centrifuge	Beckman Coulter, Inc., Brea, CA, USA
Electronic balance	Cany Precision Instrument Co., Ltd., Shanghai, China
ABI 7500 Real-Time PCR System	Applied Biosystems Life Technologies, Carlsbad, CA, USA

**Table III:** Reaction solution in the centrifuge tube.

Component of reaction solution	Dosage (μl)
Random primer (25 pmol/μl)	1
RNA	2
DEPC-dH_2_O	≤12

DEPC, diethylpyrocarbonate.

**Table IV:** Materials added to the centrifuge tube.

Component	Dosage (μl)
Reaction solution (Table III)	12
5X reverse transcription buffer	4
dNTP mixture (10 mmol/l each)	2
RNase Inhibitor (10 U/μl)	1
ReverTra Ace	1

**Table V:** Conditions for reverse transcription.

Temperature (°C)	Time (min)
30	10
42	20
99	5
4	5

**Table VI:** Primers of PDGF-A in the reaction system.

Primer	Length (bp)	Position	Tm (°C)	GC content (%)	Sequence
Forward	20	947–966	59	55	GCAGTCAGATCCACAGCATC
Reverse	22	1001–1022	60	45	TCCAAAGAATCCTCACTCCCTA

Tm, melting temperature; GC, guanine-cytosine; PDGF, platelet-derived growth factor.

**Table VII:** Primers of PDGF-B in the reaction system.

Primer	Length (bp)	Position	Tm (°C)	GC content (%)	Sequence
Left	20	1515–1534	60	55	CTGGCATGCAAGTGTGAGAC
Right	19	1603–1621	60	53	CGAATGGTCACCCGAGTTT

Tm, melting temperature; GC, guanine-cytosine; PDGF, platelet-derived growth factor.

**Table VIII:** Reaction system used for the amplifications of the target genes and the internal control (β-actin).

Component	Dosage (μl)
Sterile double-distilled water	23
SYBR^®^ Green Realtime PCR Master Mix	25
Upstream primers (10 μM)	0.5
Downstream primers (10 μM)	0.5
cDNA	1
Total volume	50

**Table IX:** Ct values of arterial PDGF-A in the case and control groups.

Group	Ct value	P-value
Case	34.38±5.80	0.001
Control	21.94±1.05	

Results are expressed as the mean ± standard deviation. PDGF, platelet-derived growth factor; Ct, cycle threshold.

**Table X:** Ct values of arterial PDGF-B in the case and control groups.

Group	Ct value	P-value
Case	33.95±5.92	0.012
Control	24.15±3.12	

Results are expressed as the mean ± standard deviation. PDGF, platelet-derived growth factor; Ct, cycle threshold.
